# Molecular Semiconductor Surfactants with Fullerenol Heads and Colored Tails for Carbon Dioxide Photoconversion

**DOI:** 10.1002/anie.201905410

**Published:** 2019-08-12

**Authors:** Marius Kunkel, Sebastian Sutter, Sebastian Polarz

**Affiliations:** ^1^ Department of Chemistry University of Konstanz Universitätsstrasse 10 78457 Konstanz Germany

**Keywords:** artificial leaves, CO_2_ utilization, fullerenes, photocatalysis, surfactants

## Abstract

The leaf is a prime example of a material converting waste (CO_2_) into value with maximum sustainability. As the most important constituent, it contains the coupled photosystems II and I, which are imbedded in the cellular membrane of the chloroplasts. Can key functions of the leaf be packed into soap? We present next‐generation surfactants that self‐assemble into bilayer vesicles (similar to the cellular membrane), are able to absorb photons of two different visible wavelengths, and exchange excited charge carriers (similar to the photosystems), followed by conversion of CO_2_ (in analogy to the leaf). The amphiphiles contain five dye molecules as the hydrophobic entity attached exclusively to one hemisphere of a polyhydroxylated fullerene (Janus‐type). We herein report on their surfactant, optical, electronic, and catalytic properties. Photons absorbed by the dyes are transferred to the fullerenol head, where they can react with different species such as CO_2_ to give formic acid.

## Graphical abstract

**Photosynthesis with soap**: Surfactants consisting of dye molecules and a polyhydroxylated fullerene self‐assemble into bilayer vesicles that mimic key functions of photosystems II and I. They can absorb photons of two different visible wavelengths, exchange excited charge carriers, and thus enable the conversion of CO_2_ into formic acid.

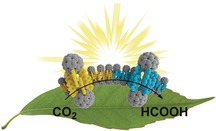

Nature has found a unique way to exploit sunlight for driving biological processes: photosynthesis. The most important constituents of chloroplasts are the photosystems II and I, which convert light into chemical energy. As photosynthesis consumes the greenhouse gas CO_2_, major research efforts have been devoted to the identification of synthetic mimics. The “artificial leaf” is an illustrative expression for this field of research.^1^ Impressive success has been achieved for photocatalytic water splitting using semiconductor nanoparticles.^2^ The absorption of light leads to the formation of electron–hole pairs, which are separated and, in an ideal case, induce photo‐reduction and ‐oxidation in one system. Numerous systems have been evaluated for the photoreduction of CO_2_, which are mostly based on inorganic semiconductors combined with suitable photosensitizers.^3^

However, as the customizability of inorganic semiconductors is restricted, it has also been considered to let molecular systems do the job.3c, 4 Fullerene derivatives have proven to be valuable compounds in optoelectronic or photocatalytic applications.^5^ Fullerenes in general have a high electron affinity, which makes them suitable for donor–acceptor systems. A common example are fullerene dyads.^6^ Fullerene dyads belong to the fascinating class of so‐called small‐molecule semiconductors,^7^ which became of interest in bulk heterojunction solar cells as strong optical absorbents and electron mediators.^8^ A donor unit is attached to the fullerene, which can be excited by absorbing light and then transfers an electron in a process accompanied by charge separation. During this process, a fullerene radical anion is produced, which can further transfer the electron.6f, 9 Studies have also shown that fullerene dyads can produce significant amounts of reactive oxygen species (ROS).^10^

Unfortunately, the use of fullerene dyads in aqueous systems is difficult because of their hydrophobic character. This problem could be addressed by a small‐molecule, fullerene‐based semiconductor with surfactant properties. Surfactants are functional molecules composed of a hydrophobic chain (typically alkyl groups) and a hydrophilic head group that are attached to each other in a dipolar fashion. The two important features of surfactants are their abilities to stabilize interfaces and to self‐assemble into higher organized structures such as micelles, vesicles, or lyotropic phases depending on concentration, temperature, and, last but not least, molecular shape. The advantage of a self‐organized superstructure formed by a molecular semiconductor surfactant is that it could come close to a new type of an artificial leaf (see Figure 1). It undergoes multi‐wavelength light triggered charge generation and separation to compartments at the two sides of an interface, followed by coupling to chemical conversion of reagents such as CO_2_ or others into different, more valuable products.3a, 11

**Figure 1:**
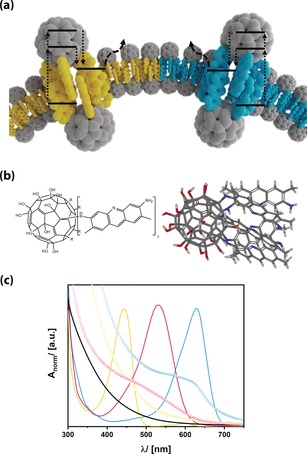
a) Fullerenol dyads as surfactant semiconductors with lipid‐like properties and associated photochemical processes. b) Schematic structure (left) and calculated structure of **FuDy‐Y**. C gray, H white, N blue, O red. c) UV/Vis absorption spectra of the dyes (lines) and the fullerenol dyads (symbols) according to the color of the dye. yellow‐G: yellow, neutral red: red, toluidine blue: blue, reference fullerenol: black.

To realize such surfactant properties, we chose a so‐called fullerenol as the head group as they are known to show similar electronic behavior as unmodified fullerenes and to be water‐soluble.^12^ We have recently presented surfactants with fullerenol head groups and alkyl tails and tested their biocompatibility.^13^ In Ref. 14, we presented a new and efficient one‐pot approach for the preparation of arbitrary Janus‐type substituted fullerenols.^14^ Whereas Ref. 14 focussed on synthetic details and molecular characterization of the compounds, any special, functional properties of those fascinating compounds have been omitted, and are the subject of the current paper.

We focused on compounds with one hemisphere of the fullerenol modified by five dye molecules (see Figure 1 a, b and Figure S1 in the Supporting Information). These dyes are acridine yellow‐G (*λ*_max_=445 nm; **FuDy‐Y**), neutral red (*λ*_max_=530 nm; **FuDy‐R**), and toluidine blue O (*λ*_max_=629 nm; **FuDy‐B**). The dyes were selected to cover almost the entire visible range (Figure 1 b). Because of their amphiphilic structure, the presented compounds are designated for possessing amphiphilic properties. Surface activity was probed by concentration‐dependent surface‐tension γ measurements (Figure 2 a). The curves show a shape characteristic for surfactants. Above a certain concentration, aggregates form. The size of those aggregates is around 100 nm for **FuDy‐Y** according to dynamic light scattering (DLS; Figure 2 b). Considering the fact that the diameter of a single surfactant molecule is only about 1.5 nm, the aggregate size cannot correspond to spherical micelles, which are expected to have double the surfactant length.

**Figure 2:**
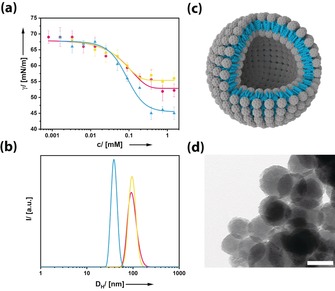
a) Concentration‐dependent surface tension measurements and b) aggregate size distribution functions derived from DLS at *c=*0.4 mm for the three different FuDy surfactants. **FuDy‐B**: blue, **FuDy‐Y**: yellow, **FuDy‐R**: red. c) Schematic structure of the vesicular aggregates formed in solution. d) TEM image of a dried sample of a colloidal solution of **FuDy‐Y** aggregates; scale bar: 100 nm.

The latter could be confirmed by transmission electron microscopy (TEM), also under cryogenic conditions, as shown in Figure 2 d (see also Figure S2). Spherical objects with diameters corresponding well to the DLS results were observed. Thus we concluded that vesicles rather than micelles had been formed with a critical aggregation concentration of *c*_cac_≈0.4 mm. Because of the relatively large packing parameter of the surfactants (see Figure S1), they behave rather similar to lipids and prefer structures of lower curvature. This observation is in line with our previous findings for fullerenol surfactants with simple alkyl chains as hydrophobic tails.^13^ Although the sizes, shapes, and polarities of the three compounds are similar according to molecular geometry optimization (Figure S1), **FuDy‐B** gave a lower γ value at saturation of the interface (*c*>0.5 mm) than **FuDy‐Y** and **FuDy‐R**. The aggregates of the latter two compounds are also larger with a hydrodynamic diameter *D*_H_ of about 90–100 nm. The different substituents in the hydrophobic, conjugated π‐system obviously have an effect on the so‐called hydrophilic–lipophilic balance (HLB).

Having shown that the **FuDys** motifs have surfactant properties, we investigated whether they are molecular semiconductors. Therefore, a thorough photophysical characterization was necessary. Optical absorption spectra of reference compounds (the unmodified dyes and C_60_(OH)_24_)^15^ are compared to each other in Figure 1 b (see also Figure S3). The absorption spectra of the different **FuDy** compounds are not simple superpositions of the spectra of their constituents.6f, 9a Instead of distinct absorption bands, an absorption edge has emerged, which is rather typical for semiconductors. The shift of the energy of the absorption edge compared to *λ*_max_ of the dyes indicates that there is electronic communication between the surfactant's head and tail. The fact that the dyes are not electronically isolated was confirmed by DFT calculations (Figure 3 a). The highest occupied molecular orbital (HOMO) is located exclusively on the dye molecules. The absolute energy of the HOMO of **FuDy‐Y**, for instance, is −5.8 eV according to photoelectron spectroscopy on air (PESA; Figure S3). The first unoccupied orbital with sufficient orbital overlap between head and tail is located at ca. −2.8 eV, and corresponds well to the optical transition (Figure 3 a). However, the DFT calculations show that the lowest unoccupied molecular orbital (LUMO) is located at −3.4 eV and consists only of fullerenol orbitals. Therefore, after electronic excitation, the charge carriers are quickly transferred to the LUMO. This was confirmed by photoluminescence (PL) measurements (Figure S3). The red‐shift in the PL maximum points to an extended conjugation length of the π‐system. However, the PL intensity is reduced by almost 90 %. Considering that fullerenols exhibit no fluorescence in the relevant spectral region (Figure S2), the decrease in the PL intensity can be interpreted as a sign for the transfer of photogenerated charges to the head group. These findings are in agreement with literature on standard fullerene dyads.^6^ Our conclusions were further confirmed by fluorescence lifetime *τ*_PL_ measurements (Figure S3). The *τ*_PL_ of **FuDy** (1.7 ns) is tremendously reduced compared to that of the free dye (*τ*_PL_=6 ns) in solution. Furthermore, the fluorescence decay of **FuDy** is not mono‐exponential anymore, which indicates that relaxation processes have become more complex upon attachment to the fullerenol residue. Further confirmation of a true semiconductor nature was obtained by current–voltage (IV) measurements of **FuDy‐Y** (Figure 3 b). The material obviously shows macroscopic charge transport, but the behavior is non‐ohmic, which is consistent with a semiconducting electronic system. Light absorption should increase the number of mobile charge carriers in a semiconductor material, and accordingly, also **FuDy‐Y** displays a signal when used as a photoconductor (Figure 3 c). After the light is switched off, the photocurrent decreases again, as expected.

**Figure 3:**
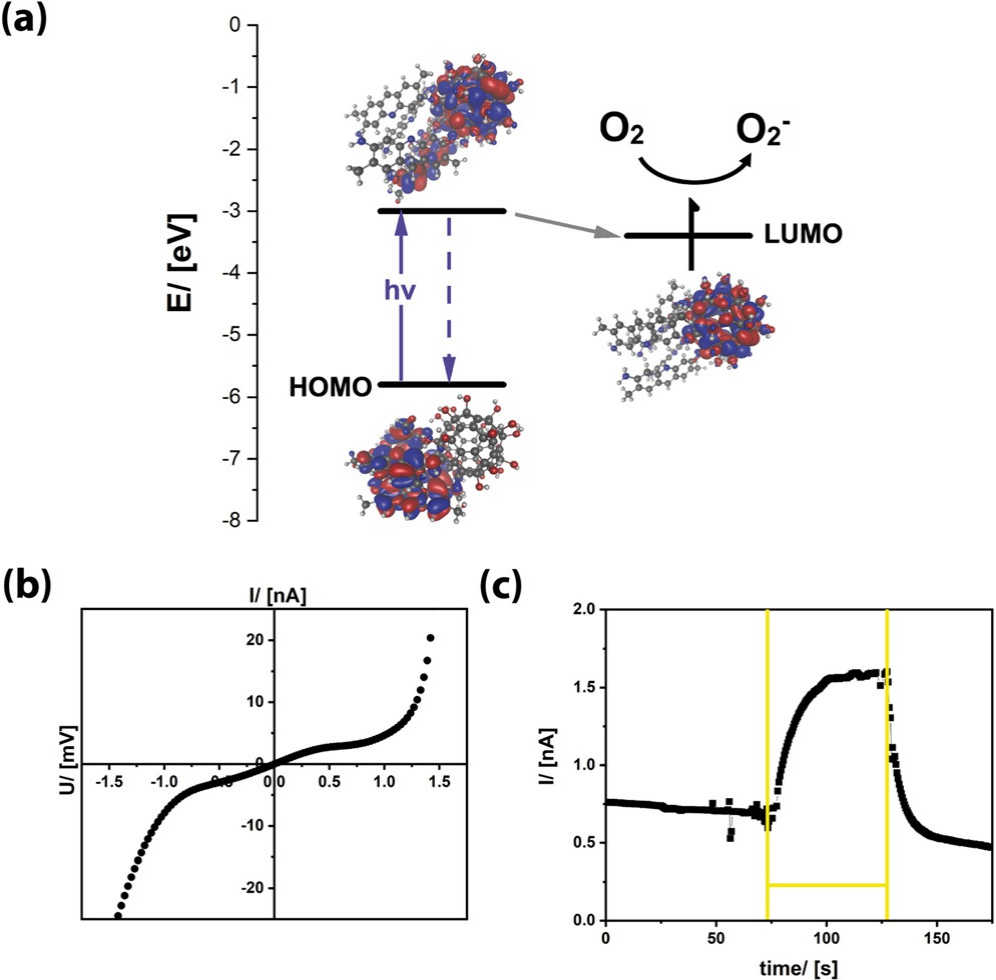
a) Frontier orbitals of **FuDy‐Y** deduced from DFT calculations and PESA measurements (HOMO: combined HOMO to HOMO−4). b) *I*/*V* measurement (measurement in the dark with 20 mV s^−1^) and c) photocurrent measurement of **FuDy‐Y** (the yellow lines mark the irradiation time with white light; 1 mV was applied).

We now expected that the charge‐separated state with the high‐energy electron depicted in Figure 3 can act as a donor state for initialization of further reactions. Thus we selected a reagent with high electronegativity, and thus energetically low‐lying acceptor orbitals, first: molecular oxygen O_2_. For quantification of the resulting superoxide, a nitrotetrazolium blue essay (NBT) was applied.10d, 16 To exclude unintentional and direct excitation of the fullerenol head group (by absorption in the UV range), tests were performed with light‐emitting diodes (LEDs) as the light source with wavelengths strictly above 400 nm (Figure S4). As only the absorption edges of **FuDy‐Y** and **FuDy‐B** correlate well with the LED, **FuDy‐R** will not be considered in the following. As a reference and for further confirmation, a non‐substituted fullerenol C_60_(OH)_24_ 15 was used. All systems containing fullerenols produced superoxide over time (Figure 4 a), but at the same concentration of the photocatalyst (*c*_cat_), both **FuDy** species produce up to 700 % more than the reference system. This result clearly demonstrates the importance of the dye entities attached to the fullerene head and the charge separation process depicted in Figure 3. Higher superoxide production can, of course, be managed by increasing the concentration of the photocatalyst (Figure 4 b). An interesting question is how a system behaves that contains two different photosystems/dyes like a leaf. Therefore, we combined **FuDy‐B** and **FuDy‐Y** under otherwise constant conditions. It does not make a difference if one uses a mixture of **FuDy‐B** and **FuDy‐Y** directly or combines aggregates of the two prepared in two separate vials. This can be understood by realizing that surfactant aggregates are highly dynamic systems in which molecules are rapidly exchanged.^17^

**Figure 4:**
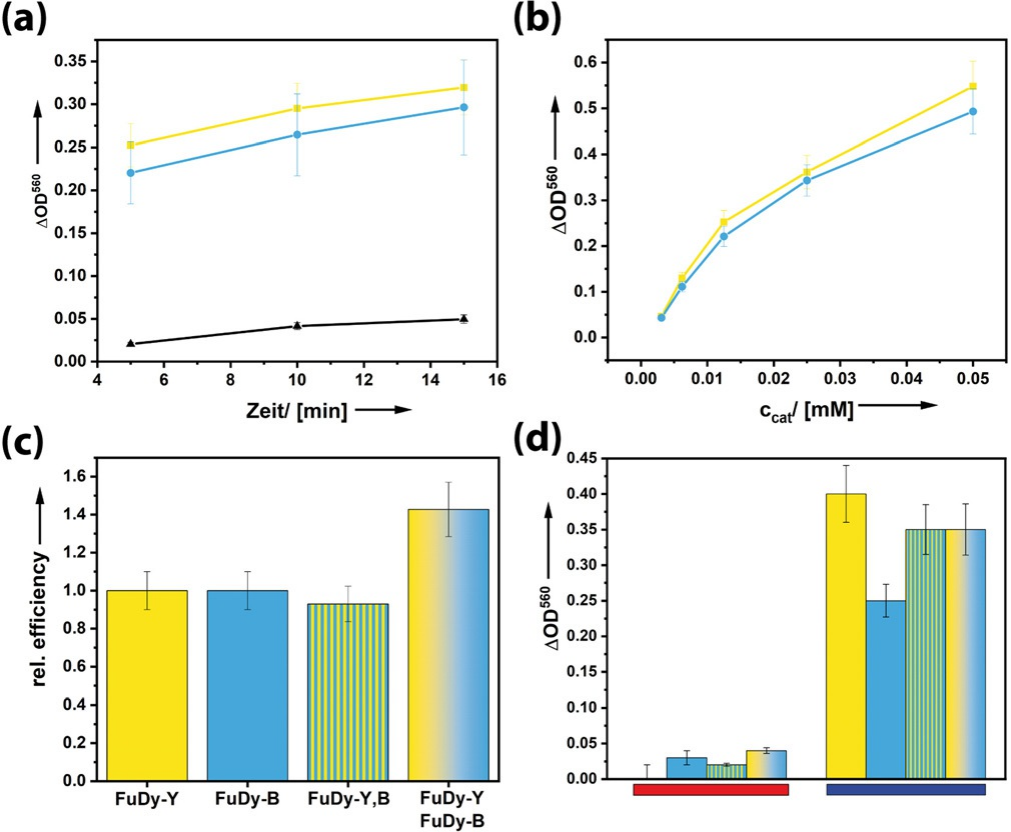
a–c) Results of the NBT essay for investigating the superoxide formation efficiency of different **FuDy** systems; C_60_(OH)_24_ (reference system): black; **FuDy‐Y**: yellow; **FuDy‐B**: blue, under white light irradiation. a) Superoxide formation over time for *c*_cat_=0.0125 mm. b) Concentration dependence; *t=*5 min. c) Comparison of the relative photocatalytic efficiencies of systems comprising only one type of dye with a mixture of **FuDy‐B,Y** (gradient color) and a **FuDy** compound containing two dyes in one molecule, *c*_cat_=0.05 mm. d) Wavelength‐dependent superoxide efficiency for irradiation with monochromatic light (red: 630 nm, blue: 450 nm) for *c*_cat_=0.05 mm.

If the two photosystems act independently from each other, one would not expect a significant change in superoxide production efficiency because the previous experiments have shown **FuDy‐B** and **FuDy‐Y** are almost equally effective (Figure 4 a, b). However, we measured an efficiency increase of 40 % (Figure 4 c). To make sure that this increase is really due to an intermolecular, cooperative effect (as in the natural leaf), we prepared a new **FuDy** with two types of dye (yellow‐G and toluidine blue) attached to the fullerenol head group in one molecule (**FuDy‐B,Y**). The efficiency of this system is slightly lower than that of the pure **FuDy** (Figure 4 c), but within the measurement error. We interpret our findings as follows. The fullerenol head group can only host one electron transferred from one of the dye molecules attached to it, followed by superoxide formation. Therefore, attaching different types of dyes to one fullerenol head does not provide any advantages. However, it seems that the **FuDy** molecules can, in their self‐assembled structures, transfer the photoexcited electron to neighboring surfactants, which then leads to an overall increase in efficiency. Furthermore, the efficiency of superoxide production at different wavelengths was evaluated. Figure 4 d shows that the **FuDy** systems, the combined as well as the mixed dyes, only produce little superoxide under irradiation at longer wavelengths whereas the efficiency tremendously increased under irradiation with blue light. As it was ensured that the fullerenol head group does not absorb in this region, this effect only derives from the absorption of the attached dyes into higher LUMOs, which then populate the charge‐separated state. These findings indicate that all absorbed wavelengths take part in the generation of superoxide whereby especially the absorption of higher‐energy photons leads to the charge‐separated state. Again, the effect of an intermolecular electron transfer can be observed.

The final, exciting question is whether the acceptor orbitals of CO_2_ are also low enough in energy for undergoing the described photoreduction process. Thus the experiments were repeated with CO_2_ instead of O_2_. The results were evaluated by ^1^H NMR spectroscopy (Figure 5 a) and GC‐MS. We clearly observed the generation of formic acid when **FuDy‐B/FuDy‐Y** was used as the photocatalyst. Formic acid can be formed by a two‐electron two‐proton reaction, which is likely to happen directly at the **FuDy**’s head groups. Figure 5 b, c shows a proposed mechanism for this process.^18^ We assume that the general mechanism is similar to that of photocatalyzed superoxide production of fullerenes and fullerenols, in which the fullerenol is likely to transfer electrons.10b, 10d, 12a, 19 The reducibility of the fullerenol compound strongly depends on the degree of polyhydroxylation and the type of polyhydroxylation moieties and can vary between a slightly negative or a slightly positive potential, whereas a stronger reducibility derives from a larger π‐electron stabilization.^20^ An intramolecular charge transfer results in a reduction of the head group, whereas the charge is located at the C_60_ core as oxygen has a weak ability to accommodate radical electrons.^20^ This fullerenol anion, according to the known literature mentioned above, can reduce, for example, carbon dioxide in a fast process to again enable the reduction of the fullerenol. The fullerenol head group is predestined for the coordination of, for example, carbon dioxide because of the multiple hydrogen binding sites. In a first step, carbon dioxide is reduced to yield the carbon dioxide radical anion. After protonation at the carbon atom, a fast second electron transfer has to take place, which emphasizes the importance of the intermolecular exciton transfer described before. Final protonation yields formic acid. No further reduction occurs according to GC‐MS. Performing the reaction at different pH values shows that the highest yield of formic acid was achieved at pH 7, with decreasing efficiency with decreasing pH value. The yield of formic acid at pH 5 is only about 18 % of the value at pH 7, whereas at more acidic pH values, almost no formic acid is produced. At pH 7, the predominant species is HCO_3_^−^, which can obviously interact very well with the fullerenol surfactants.

**Figure 5:**
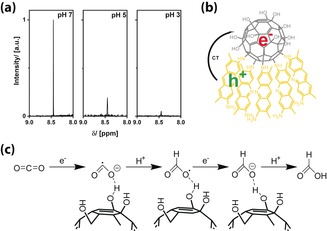
Photoreduction of carbon dioxide with a mixture of **FuDy‐Y** and **FuDy‐B** in aqueous solution. a) Excerpts of ^1^H NMR spectra for formic acid production from CO_2_ at different pH values. b) Overall scheme for the **FuDy**‐catalyzed reaction. c) Proposed mechanism for the photoreduction of CO_2_ by the **FuDy** system (water molecules not shown).

The leaf is an unparalleled example for the “green” conversion of energy in the form of light into valuable products. Therefore, numerous attempts have been made in materials science to create systems with similar functionality. We have presented a new approach based on multifunctional surfactants. Inspired by literature on fullerene dyads and our own work on amphiphiles with fullerenol head groups, we have discussed surfactants with semiconductor properties. The compounds self‐assemble into bilayer vesicle structures in solution and exhibit properties such as charge separation of photogenerated excitons. Intermolecular electron transfer takes place between surfactant fullerenol dyads covering different regions of the electromagnetic spectrum, thus fulfilling one key criterium of an artificial leaf. The chemical energy of the charge‐separated states could be exploited to produce superoxide from oxygen and formic acid from carbon dioxide by irradiation with visible light in aqueous solution. The dependence of the efficiency on different parameters was evaluated, and a mechanism was proposed. Therefore, also the second criterion of an artificial leaf, the conversion of less valuable compounds (CO_2_) into better products, has been realized. The current work extends not only the horizon of functional fullerene‐based materials but also that of surfactant chemistry in general.

## Conflict of interest

The authors declare no conflict of interest.

## Supporting information

As a service to our authors and readers, this journal provides supporting information supplied by the authors. Such materials are peer reviewed and may be re‐organized for online delivery, but are not copy‐edited or typeset. Technical support issues arising from supporting information (other than missing files) should be addressed to the authors.

Supplementary
