# Supplementary material for: Molecular Semiconductor Surfactants with Fullerenol Heads and Colored Tails for Carbon Dioxide Photoconversion
Source: Angew Chem Int Ed Engl. 2019 Aug 12;58(44):15620–5. doi: 10.1002/anie.201905410 (PMC6851540; doi:10.1002/anie.201905410)
Supplement: Supplementary file 1 — Supplementary [file ANIE-58-15620-s001.pdf]

Angewandte Chemie
Eine Zeitschrift der Gesellschaft Deutscher Chemiker

Supporting Information

Molecular Semiconductor Surfactants with Fullerenol Heads and Colored Tails for Carbon Dioxide Photoconversion

*Marius Kunkel, Sebastian Sutter, and Sebastian Polarz**

anie_201905410_sm_miscellaneous_information.pdf

SUPPORTING INFORMATION

____

Experimental part.

The synthesis was performed according to a protocol by Kunkel et al.[1] For the synthesis of **FuDy-Y,B** a mixture of acridine yellow and toluidine blue was used instead of only one dye. This result is a statistic mixture. Additionally, to the protocol the compounds have been purified by HPLC (see Analytical methods).

The yield of the compounds is: FuDy-Y 60 %, FuDy-R 69 %, FuDy-B 45 %, FuDy-Y,B 38 %.

Characterization

FuDy-Y

^{1}H-^{13}C- CP MAS-NMR (100 MHz): δ (ppm) 176, 160, 133, 77, 56, 39, 15

ATR-IR: ν (cm^{-1}) = 3200, 2927, 2874, 2607, 1564, 1372, 1244, 989, 818

TGA: 40-192 °C 11 %; 192-550 °C 17 %; 550 °C+ 72 % → 20 +/- 2 OH

ESIMS: $[(OH)_8O_{12}C_{60}(C_{15}H_{14}N_3)_5]^+$ m/z = 2230.2 (2230.2); $[(OH)_4O_{17}C_{60}(C_{15}H_{14}N_3)_5]^-$ m/z = 2242.2 (2242.2)

FuDy-R

^{1}H-^{13}C- CP MAS-NMR (100 MHz): δ (ppm) 177, 160, 150, 137, 129, 101, 74, 57, 39, 18

ATR-IR: ν (cm^{-1}) = 3177, 2957, 2858, 1580, 1443, 1359, 1002

TGA: 40-180 °C 11 %; 180-575 °C 22 %; 575 °C+ 67 % → 21 +/-3 OH

ESIMS: $[(OH)_{17}O_4C_{60}(C_{15}H_{15}N_4)_5]^+$ m/z = 2330.3 (2330.3); $[(OH)_{17}O_3C_{60}(C_{15}H_{15}N_4)_5]^-$ m/z = 2314.3 (2314.3)

FuDy-B

^{1}H-^{13}C- CP MAS-NMR (100 MHz): δ (ppm) 176, 160, 133, 79, 57, 39, 15

ATR-IR: ν (cm^{-1}) = 3200, 2927, 2874, 1564, 1361, 1010

TGA: 40 – 200 °C 10%; 200-570 °C 21 %; 570 °C+ 69% → 20+/-3 OH

ESIMS: $[(OH)_{10}O_{11}C_{60}(C_{15}H_{16}N_3S)_5]^+$ m/z = 2418.6 (2418.6); $[(OH)_{16}O_4C_{60}(C_{15}H_{16}N_3S)_5]^-$ m/z = 2408.6 (2408.6)

FuDy-Y,B

ATR-IR: ν (cm^{-1}) = 3231, 2943, 2893, 1560, 1342, 1169, 1011

ESIMS: $[(OH)_{14}O_8C_{60}(C_{15}H_{14}N_4)_2(C_{15}H_{16}N_3S)_3]^+$ m/z = 2370.5 (2370.5)

Methods
General Methods
Synthesis that acquired inert gas atmosphere were performed using general Schlenk techniques under argon atmosphere. The solvents were dried according to standard literature and stored under argon. Water was deionized with Millipore Milli-Q. All starting materials used for synthesis were purchased from commercial sources unless stated differently. The fullerene C_{60} (pur. 99.9 %) was purchased from Research & Production Company “Modern Synthesis Technology”.
Analytical Methods
Liquid chromatography was measured with Thermo Fisher Scientific Dionex 3000. As the column, Agilent Poroshell 120 EC-C18 (2.1×100 mm, 2.7µm) was used. MeCN (5%) as eluent A and 95% water as eluent B with 0.1% formic acid were used. A linear gradient of 5% A to 100% A was applied a flow rate of 0.3 mL/min. Mass spectra were measured on Bruker amazon SL respectively on Bruker microtof II system in pos. or neg. mode via direct inject from a methanolic solution. Assignment of peaks was done as follows: $[M - xH_2O - yH - zO - v(HNR)]^{a-/+}$. Molecular ion peak is identified in combination with results from TGA and NMR. TGA was measured on Netzsch Jupiter STA 449 F3. All measurements were performed under nitrogen atmosphere with 80 mL/min flowrate and with heating rate of 5 K/min. MAS-NMR measurements were performed on Bruker Avance II 400 solid-state NMR at 295 or 350 K and with 10k Hz rotational speed. Number of scans 5k – 10k with D1 = 10 to 100 sec. NMR measurements (^{1}H, ^{13}C) were performed on a Varian INOVA 400 MHz spectrometer. Attenuated total reflection−infrared (ATR−IR) spectra were measured with a Perkin Elmer100 Spectrum spectrometer including an ATR unit. DLS measurements were done by using a Malvern Zen5600. Transmission electron microscopy (TEM) observations were carried out using Zeiss Libra120. For cryo transmission electron microscopy studies, a sample droplet of 2ul was put on a lacey carbon filmed copper grid (Science Services, Muenchen), which was hydrophilized by air plasma glow discharge unit (30s with 50W, Solarus 950, Gatan, Muenchen, Germany). Subsequently, most of the liquid was removed with blotting paper in a Leica EM GP (Wetzlar, Germany) grid plunge device, leaving a thin film stretched over the lace holes in the saturated water atmosphere of the environmental chamber. The specimens were instantly shock frozen by rapid immersion into liquid ethane cooled to approximately 97K by liquid nitrogen in the temperature-controlled freezing unit of the Leica EM GP. The temperature was monitored and kept constant in the chamber during all the sample preparation steps. The specimen was inserted into a cryotransfer holder (CT3500, Gatan, Muenchen, Germany) and transferred. Surface tension measurements were performed at Krüss K100.
Photosensor measurements were performed on 3 mm×3 mm sensor substrates from Umweltsensortechnik and measured with a Zahner IM6 potentiostat. Measurements in solution were performed in a folded capillary cell. UV/Vis absorption spectra were acquired using an Agilent Cary 60 spectrometer. PL spectra were obtained from a PicoQuant FluoTime-300 spectrometer. Geometry optimization and frontier orbital calculation was performed using Density-Functional Theory (DFT) with the TURBOMOLE Program Package for ab initio Electronic Structure Calculations using B3LYP/def2-TZVP level of theory. TURBOMOLE V7.1, a development of University of Karlsruhe and Forschungszentrum Karlsruhe GmbH, 1989-2007, TURBOMOLE GmbH, since 2007; available from http://www.turbomole.com.

NBT assay
0.5 mM NBT and 0.05/0.025/0.0125/0.0061 mM FuDy in Milli-Q were irradiated for 5/10/15 min with 100 W white LED (see Fig. S3). The difference in absorbance was measured at 560 nm versus blank sample.

CO_2 conversion
5mM aqueous solution (pH 7, pH 5 and pH 3) of FuDy-Y,B has been saturated with 100% CO_2 gas. The vessel was sealed and irradiated with 100 W white LED (see Fig. S3). Formic acid was detected with ^{1}H-NMR. 0.1 M solution of ascorbate was added as electron donor.

Figure S1. Molecular structure of FuDy-compounds.
(a) Lewis-structures.

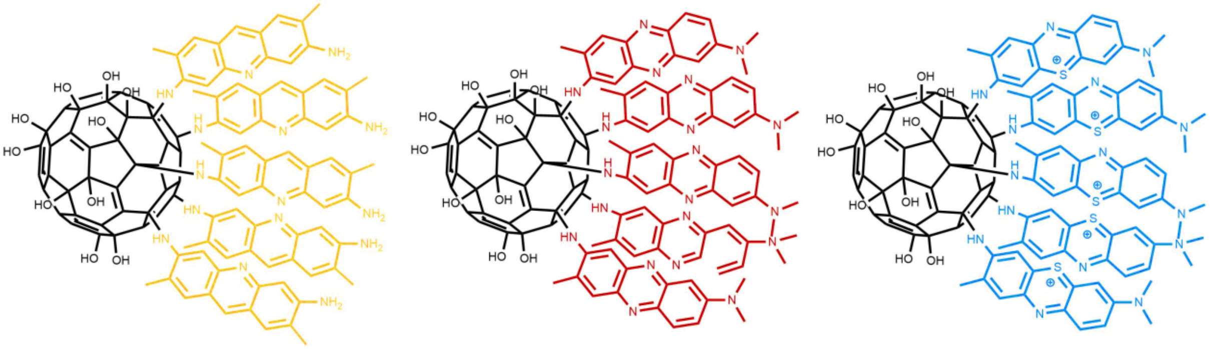

FuDy-Y **FuDy-R** **FuDy-B**

(b) Calculated molecular geometry and electrostatic potential maps.

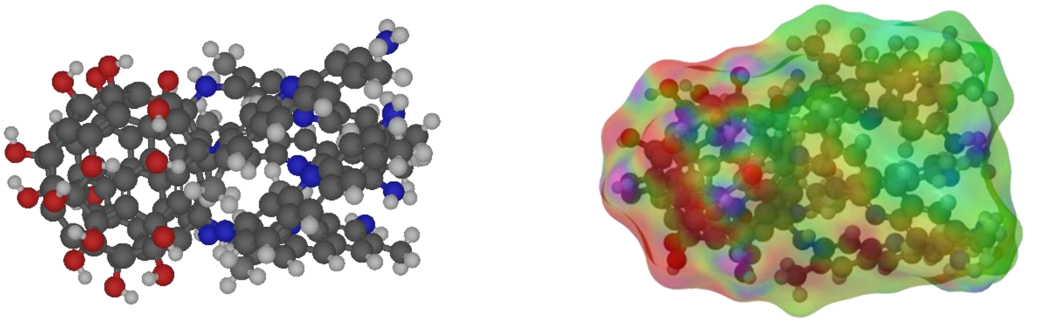

FuDy-Y

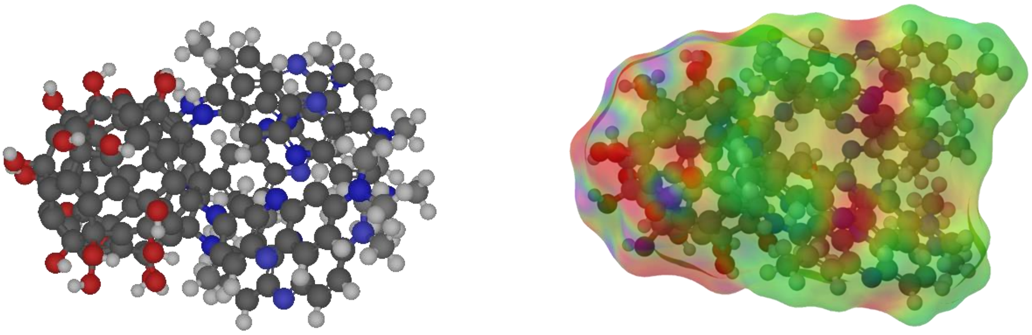

FuDy-R

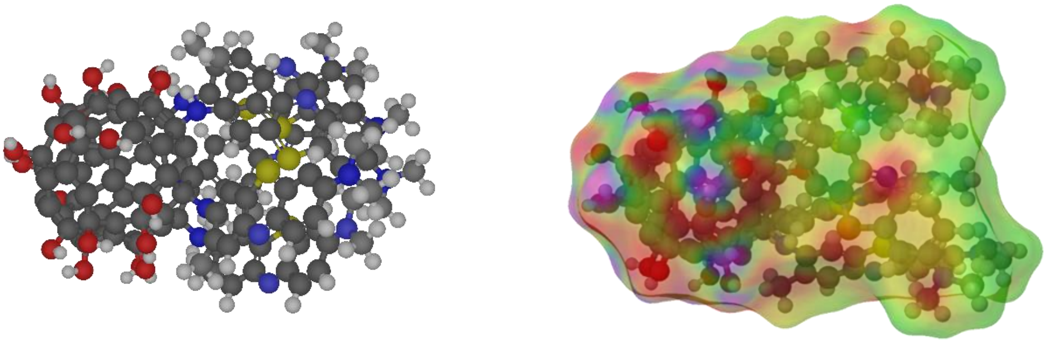

FUDY-B

Figure S2. Cryo-TEM investigation of FuDy-Y aggregates in solution.

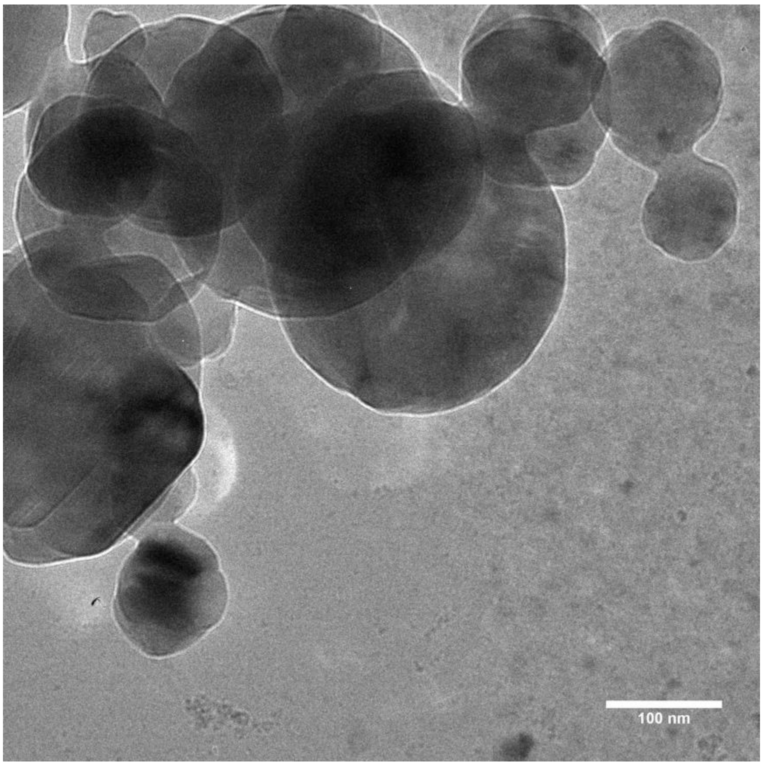

Figure S3. Photophysical characterization of **FuDy** compounds.

(a) Quantitative adsorption spectra.

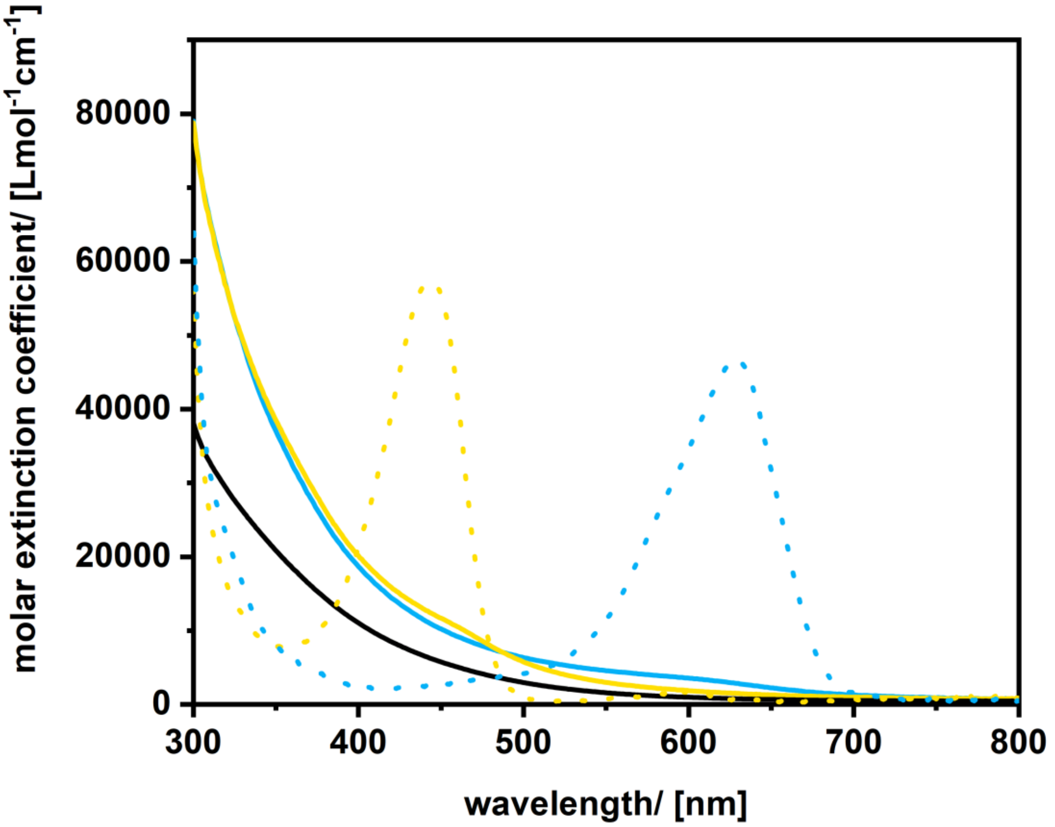

yellow dotted = yellow-G, blue dotted = toluidine blue, black = $C_{60}(OH)_{24}$, yellow line = **FuDy-Y**, blue line = **FuDy-B**.

(b) PL spectra.

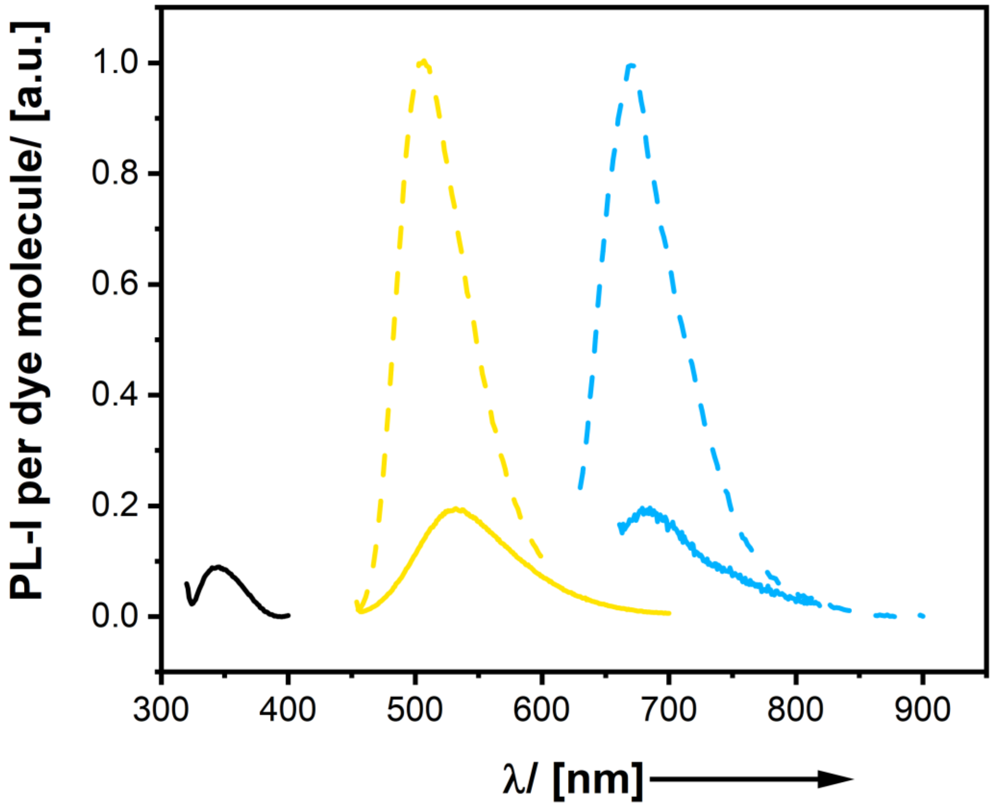

blue dashed = yellow-G, red dashed = toluidine blue, black = $C_{60}(OH)_{24}$, blue line = **FuDy-Y**, red line = **FuDy-B**.

(c) PESA

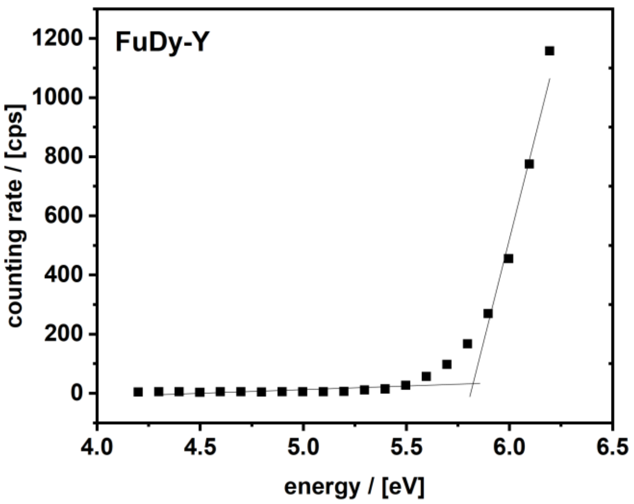

FuDy-Y
counting rate / [cps]
1200
1000
800
600
400
200
0
4.0
4.5
5.0
5.5
6.0
6.5
energy / [eV]

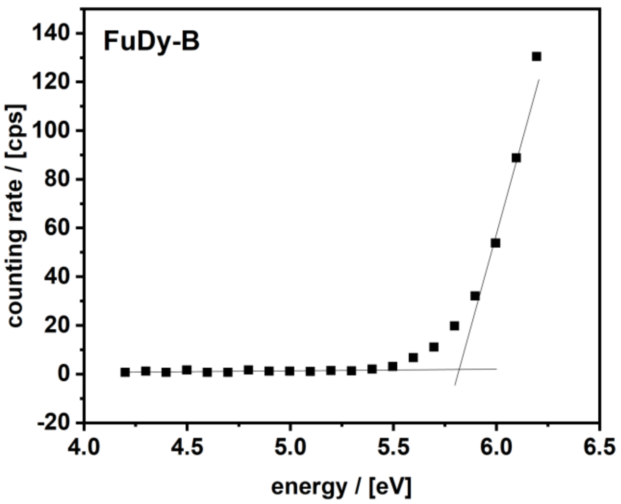

FuDy-B
counting rate / [cps]
140
120
100
80
60
40
20
0
-20
4.0
4.5
5.0
5.5
6.0
6.5
energy / [eV]

(d) fluorescence lifetime

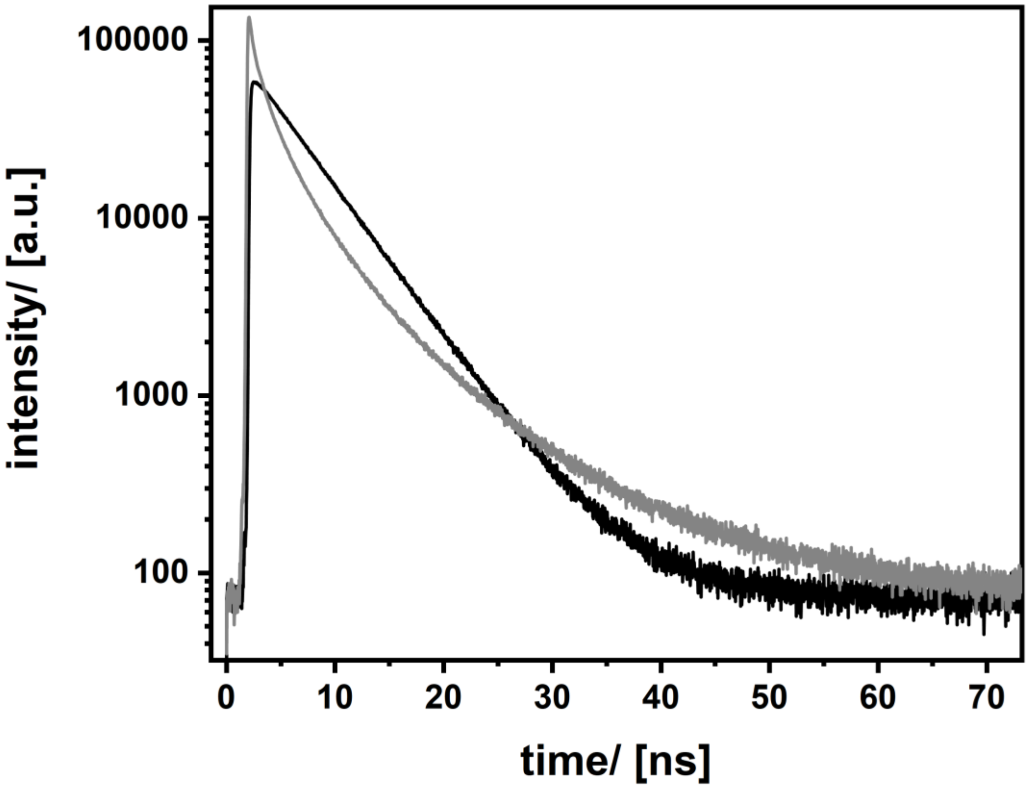

Figure S4. Spectrum of the white LED used for the photochemical reactions.

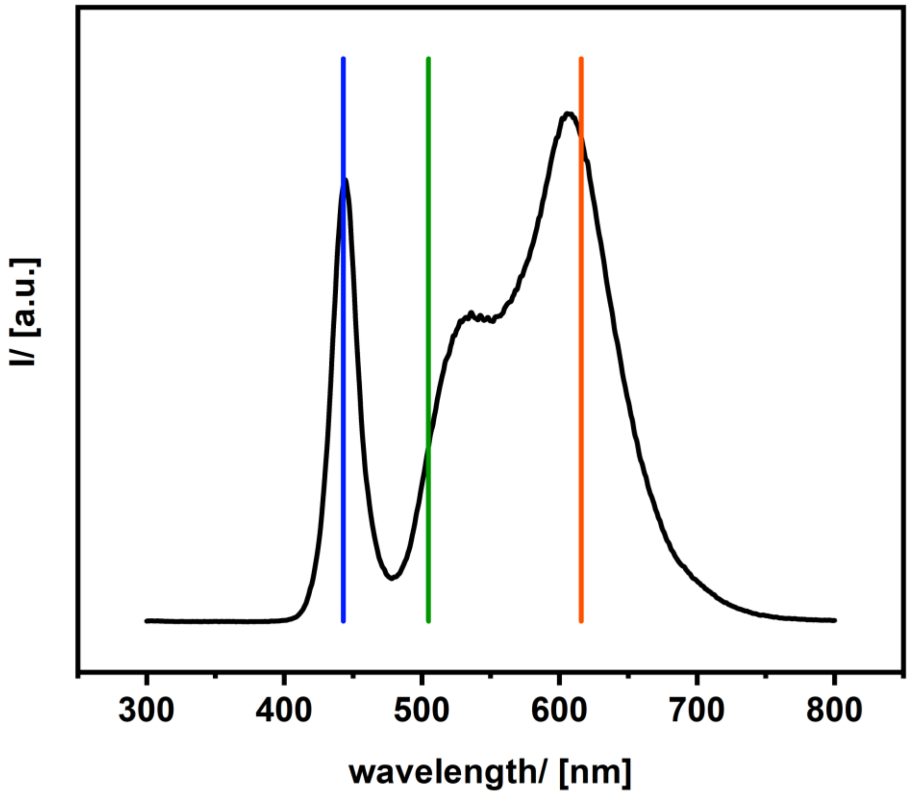

The vertical bars show the position of the absorption maxima of the dyes used in the current study.

References

1. Kunkel, M.; Polarz, S., Easy, efficient and versatile one-pot synthesis of Janus-type-substituted fullerenols. *Beilstein Journal of Organic Chemistry* **2019,** *15*, 901-905.
